# The Role of *Pseudomonas aeruginosa* Lipopolysaccharide in Bacterial Pathogenesis and Physiology

**DOI:** 10.3390/pathogens9010006

**Published:** 2019-12-19

**Authors:** Steven M. Huszczynski, Joseph S. Lam, Cezar M. Khursigara

**Affiliations:** Department of Molecular and Cellular Biology, University of Guelph, 50 Stone Rd E, Guelph, ON N1G 2W1, Canada; shuszczy@uoguelph.ca (S.M.H.); jlam@uoguelph.ca (J.S.L.)

**Keywords:** lipopolysaccharide, O antigen, host–pathogen interactions, cystic fibrosis, biofilms, antimicrobial resistance, pyocin

## Abstract

The major constituent of the outer membrane of Gram-negative bacteria is lipopolysaccharide (LPS), which is comprised of lipid A, core oligosaccharide, and O antigen, which is a long polysaccharide chain extending into the extracellular environment. Due to the localization of LPS, it is a key molecule on the bacterial cell wall that is recognized by the host to deploy an immune defence in order to neutralize invading pathogens. However, LPS also promotes bacterial survival in a host environment by protecting the bacteria from these threats. This review explores the relationship between the different LPS glycoforms of the opportunistic pathogen *Pseudomonas aeruginosa* and the ability of this organism to cause persistent infections, especially in the genetic disease cystic fibrosis. We also discuss the role of LPS in facilitating biofilm formation, antibiotic resistance, and how LPS may be targeted by new antimicrobial therapies.

## 1. Introduction

**Figure 1:**
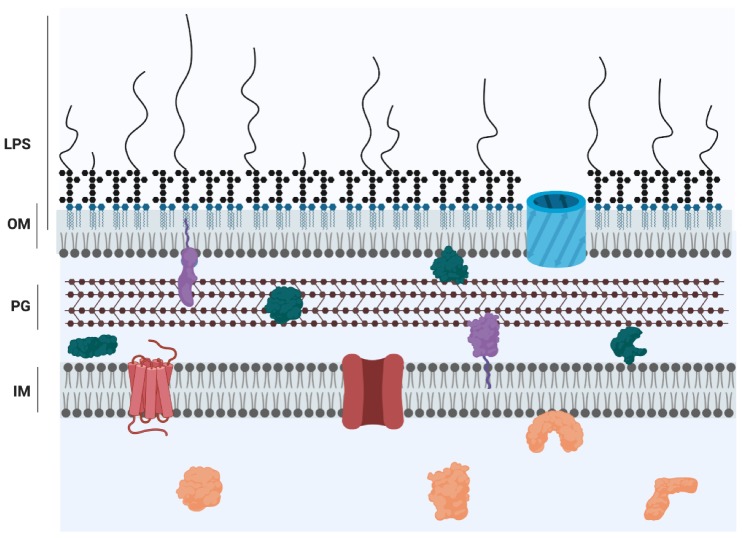
Cartoon representation of the Gram-negative cell envelope. The inner membrane (IM) is a symmetric bilayer comprised of phospholipids while the outer membrane (OM) is an asymmetric bilayer containing phospholipids in the inner leaflet and LPS in the outer leaflet. The domains of LPS are represented as follows: lipid A, blue; core, black hexagons; O antigen, curved lines. The membranes are separated by the periplasmic space, which contains a thin layer of peptidoglycan (PG) [8]. Proteins are localized to all compartments and represented by the following colours: orange, cytoplasmic proteins; red, inner membrane proteins; purple, lipoproteins; green, periplasmic proteins; blue, outer membrane protein.

*Pseudomonas aeruginosa* is a Gram-negative bacterium that is a global threat to public health and is classified as one of the ESKAPE pathogens, a group of microorganisms with a high propensity for causing problematic, drug-resistant, nosocomial infections [1]. In the hospital setting, contamination of sinks, plumbing, and water are a significant reservoir for *P. aeruginosa*, and are often the source of an infection [2]. This bacterial species is versatile and can cause disease by colonizing a variety of human host sites, such as burn wounds, the urinary tract, and the respiratory system [3], but can also cause disease in plants [4,5]. *P. aeruginosa* is notorious as a significant cause of morbidity and mortality in those with cystic fibrosis (CF), an autosomal recessive genetic disorder causing ion imbalance in the lungs, which leads to a thick and sticky mucous that hinders mucociliary clearance of potential pathogens [6]. In the end-stage of the disease, *P. aeruginosa* is typically the dominant organism infecting the lung [7]. The success of *P. aeruginosa* as a pathogen is due to its intrinsic and acquired antibiotic resistance mechanisms, ability to establish robust biofilms, and repertoire of virulence factors, including a number of secreted enzymes and molecules causing extensive host tissue damage [3]. *P. aeruginosa* also expresses the major virulence factor lipopolysaccharide (LPS), which is an integral component of the archetypal cell envelope of most Gram-negative bacteria (GNB). GNB possess two membranes separated by the periplasmic milieu containing a thin layer of peptidoglycan [8]. Although there are a few exceptions, the outer membrane (OM) is an asymmetric bilayer of phospholipids on the periplasmic face and LPS on the extracellular face [9] (Figure 1). Inherent to its localization, LPS plays significant roles in interactions with the bacterium’s environment. Both the hydrophobic and polar nature of LPS contribute to a drastic decrease in membrane permeability in GNB; the lipid membrane impedes the passage of polar solutes, whereas the polar LPS groups repel lipophilic compounds [10]. LPS is conceptualized as consisting of three distinct domains: lipid A, core oligosaccharide, and O antigen (also called the O polysaccharide, O-antigen, or O-polysaccharide). These regions have both distinct and overlapping functions in bacterial physiology. This review focuses on the role of lipid A, core, and O antigen in the sensing of LPS by host defense systems, targeting by antimicrobials, and the pathogenesis of *P. aeruginosa*.

## 2. Structure of Lipid A, Core, and O Antigen

**Figure 2:**
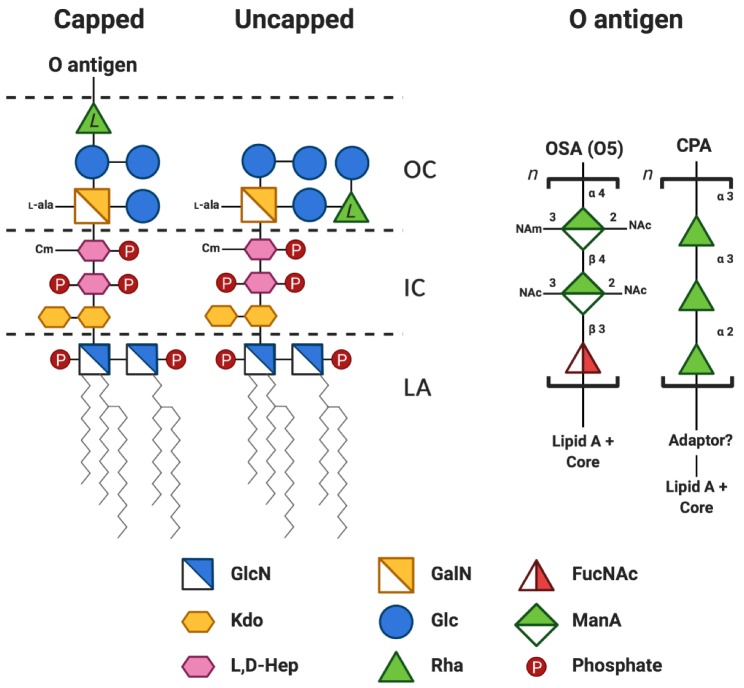
Simplified chemical structure of *P. aeruginosa* PAO1 (serotype O5) lipopolysaccharide. The structure is adapted from several studies [12,13,14,15,16,17] and coloured according to the Symbol Nomenclature for Glycans (SNFG) [18,19]. A more detailed review of the chemical structure of *P. aeruginosa* LPS can be found in [17]. The lipid A-core region can be capped (or not) with a variable number of O antigen repeats. The predominant penta-acylated lipid A structure is shown. For clarity, the following modifications to the core sugars are not shown: the phosphorylation sites on the two heptose residues are depicted as monophosphorylated but may contain mono- di- or triphosphates; non-stoichiometric *O*-acetylation of the outer core sugars; the phosphate at position 2 in Hep II is non-stoichiometrically modified with phosphoethanolamine. The l-configuration of the rhamnose in the core is denoted by l to distinguish it from d-rhamnose found in the CPA repeat unit. A short sugar adapter may be present between CPA and the lipid A-core. OC, outer core; IC, inner core; LA, lipid A; GlcN, glucosamine; GalN, galactosamine; FucNAc, *N*-acetyl-d-fucosamine Kdo, 3-deoxy-d-*manno*-oct-2-ulosonic acid; Glc, glucose; ManA, manuronic acid; l,d-Hep, l-*glycero*-d-*manno*-heptose; Rha, rhamnose; Cm, 7-*O*-carbamoylation; l-Ala, 2-l-alanylation; *n*, variable number of repeats; NAm, *N*-amidino; NAc, *N*-acetyl.

Lipid A is the hydrophobic portion of LPS that anchors the molecule in the OM. It is an acylated glucosamine disaccharide that is phosphorylated on the 1 and 4′ positions. The tight packing of lipid A in the OM constitutes a gel-like permeability barrier to small hydrophobic solutes [10]. Although the structure and synthesis of lipid A is generally conserved, the number of acyl chains, degree of phosphorylation, and presence of other modifications can vary, all of which have important implications for interactions with the bacterium’s environment or host (see below) [11]. The predominant lipid A of *P. aeruginosa* PAO1, a thoroughly studied laboratory-adapted strain, is shown in Figure 2.

The structure of the core oligosaccharide is overall more varied amongst GNB than lipid A, yet there are several conserved features. The core oligosaccharide is divided into two regions, the inner core and the outer core. The inner core typically contains Kdo (3-deoxy-d-*manno*-oct-2-ulosonic acid) covalently linked to several heptose residues (either l,d-Hep, or d,d-Hep), although some core structures contain Ko (d-*glycero*-d-*talo*-oct-2-ulopyranosonic acid) instead of Kdo, or no heptose [20]. The heptoses are the targets of modifications, such as the addition of phosphate and phosphoethanolamine. The outer core region varies among bacterial species, but typically contain hexoses and hexosamines. In *P. aeruginosa*, the core oligosaccharide is heavily phosphorylated and composed of Kdo, heptose, galactosamine, glucose, and rhamnose (Figure 2). The negative charges on the core provide membrane stability through bridging interactions with divalent cations [10] and the proper folding of some outer membrane proteins are dependent on specific protein-core interactions [21]. Indeed, in the literature, *Escherichia coli* mutants with truncations of the core oligosaccharide exhibit a number of OM defects and are particularly susceptible to hydrophobic antibiotics and anionic detergents [10,22]. However, the core is not necessarily essential for viability, since mutants of *E*. *coli* lacking any core sugars and containing only lipid IV_A_ (a tetra-acylated, di-phosphorylated, di-glucosamine) have been isolated, albeit, some in the presence of compensatory mutations [23,24,25,26].

The O antigen is the long polysaccharide component of LPS, the length of which can vary from one to hundreds of sugars. O antigen is synthesized separately from the lipid A-core and later attached to it. Consequently, not every lipid A-core molecule is appended with O antigen before export. The result is a heterogeneous OM surface containing LPS with and without O antigen (Figure 1). LPS containing lipid A-core only or lipid A-core and O antigen are both exported to the cell surface. Those LPS molecules containing O antigen are termed “capped”, while the ones lacking O antigen are termed “uncapped”. In the literature, bacteria containing O antigen-capped LPS are often described as “smooth” whereas those devoid of O antigen are termed “rough”. These terms refer to the smooth and rough colony morphologies of the bacteria when grown on solid media, rather than the properties of the bacterial membrane [27]. The O antigen polymer is comprised of repeating sugar units that are highly variable in structure between and within species [11]. In *E*. *coli* alone there are more than 180 known unique O antigen structures [28]. This variability is the basis of the intraspecies classification system, known as O-serotyping, which categorizes strains based on the specific O antigen presented on the cell surface. Serotyping of *P. aeruginosa* was originally developed using immunochemical assays but has now been supplemented with genetic methods such as PCR and sequencing. [27,29]. We guide the reader to Lam et al. [27] for a perspective on the history, benefits, and challenges of different serotyping methods in *P. aeruginosa*. The heterogeneity of O antigen structures is the result of differences in the identities of the sugars in the repeat unit, the linkages between them, the presence or absence of side branches, and non-stoichiometric modifications. *P. aeruginosa* can simultaneously produce two O antigens: the common polysaccharide antigen (CPA) and the O-specific antigen (OSA) [27,30]. CPA has a common, conserved structure, consisting of repeating units of →3)d-Rha(α1→3)d-Rha(α1→2)d-Rha(α1→ whereas the OSA structure is variable, and therefore the determinant used in serotyping to segregate this bacterial species into many groups [16,31].

## 3. Interactions of LPS with the Host Immune System

### 3.1. Animal and Plant Receptors Recognize LPS and Mount an Immune Response

The surface exposure of LPS and the conservation of certain structural features across species make it a primary elicitor of host defenses. In mammals, LPS is a microbe-associated molecular pattern (MAMP) that can be a potent activator of the host innate immune response by inducing the activation of signal transduction cascades, which invariably lead to the production of proinflammatory cytokines [32]. Over-activation of these pathways can cause the life-threatening syndrome septic shock. Extracellularly, LPS monomers are extracted from GNB or their outer membrane vesicles (OMVs) by LPS-binding protein (LBP) and transferred to soluble or membrane-bound CD14. LPS is then transferred to MD-2/TLR4 monomers, inducing dimerization and the activation of Mal/MyD88-dependent signaling. Alternatively, endocytosis of activated complexes induces TRIF-dependent signaling and a different immunological response [32]. Within the cytosol, LPS is bound directly by caspases (caspase 4 and caspase 5 in humans, caspase 11 in mice), which in turn activate GSDMD (gasdermin family protein). GSMD forms pores on the plasma membrane, which induces cell pyroptosis and facilitates the release of interleukins [33].

The main interaction of LPS with MD-2/TLR4 is through the acyl chains of lipid A, which pack within a pocket of MD-2. The C2 acyl chain protrudes from this pocket and forms a dimerization interface for a stretch of hydrophobic amino acids within TLR4. The phosphates of the GlcN residues also contribute to ionic interactions that are necessary for optimal agonism [34]. Although the interactions of inner core Kdo residues are not essential for dimerization of the MD-2_2_/TLR4_2_/LPS_2_ complex, lipid A molecules containing Kdo tend to be more potent agonists than their cognate lipid A molecules lacking Kdo [35]. In general, hypo-acylated and hypo-phosphorylated forms of lipid A are less potent agonists, or even antagonists, of TLR4 signalling [36,37,38]. However, this does not always hold true, as a clinical isolate of *Burkholderia cenocepacia* is known to be able to induce MD2-TLR4 activation (albeit to a lesser extent than *E. coli* hexa-acylated LPS) despite only expressing tetra and penta-acylated lipid A. In this case, the longer acyl chains and aminoarabinosylation of lipid A seem to compensate for this hypo-acylation [39]. The lipid A isolated from *P. aeruginosa* CF isolates are typically hexa- and hepta-acylated, which apparently are more potent in eliciting inflammatory responses compared to the pentacylated lipid A typically found in laboratory-adapted strains or those derived from non-CF-related infections. The acylation pattern is associated with disease severity since hepta-acylated lipid A is associated with late-stage CF infections [14,40,41,42].

In addition to TLR4, a number of studies have implicated another membrane receptor, the cystic fibrosis transmembrane conductance regulator (CFTR), in recognition of *P. aeruginosa* LPS. The CFTR is an important pathogen recognition molecule because it extracts LPS from the bacterial membrane and activates an inflammatory response via nuclear translocation of NF-κB [43]. The outer core oligosaccharide was identified as the ligand for CFTR and interacts with the first extracellular loop of this protein [44,45,46]. Individuals with CF are homozygous for CFTR alleles that negatively affect the transport, processing, or function of this ion channel. The most common of these alleles is the ΔF508 mutation [47]. Experiments using epithelial cell lines carrying either the wildtype or ΔF508 variants of CFTR indicated that the internalization of *P. aeruginosa* was reduced when mutant but not wildtype CFTR was expressed, suggesting that CFTR mutations may promote *P. aeruginosa* infection [44]. In contrast, CFTR-dependent internalization of *P. aeruginosa* in corneal epithelial cells is necessary for this bacterium to cause keratitis [48,49,50]. Similarly, in *Salmonella enterica* serovar Typhi, entry into intestinal epithelial cells is also mediated by CFTR, and it has been documented that CF patients possessing mutant forms of this protein might be protected from contracting typhoid fever [51].

Plants are also able to sense LPS and activate signaling pathways that ultimately lead to an innate immune response that includes reactive oxygen species (ROS) bursts, callose deposition, nitric oxide production, and transcription of defense-related genes [52,53,54]. The mechanisms underlying the recognition of LPS have only recently started to be understood. In *Arabidopsis thaliana*, a bulb-type S-domain 1 receptor-like kinase, termed LORE (lipooligosaccharide-specific reduced elicitation), was found to mediate the sensing of LPS and trigger a pathogen response. This LORE-dependent response was induced by LPS from *P. aeruginosa*, *P. syringae*, and *Xanthomonas campestris*, but not from *E. coli*, *S. enterica*, or *Burkholderia* spp. LORE was initially thought to minimally interact with lipid A, and this interaction was enhanced by the presence of the core oligosaccharide, but not the O antigen [53]. However, a follow up investigation determined that LORE senses medium chain-3-hydroxy-fatty acids (mc-3-OH-FA). Although these fatty acids are a component of *Pseudomonas* lipid A and other pseudomonal compounds, only free mc-3-OH-FA is sensed by LORE. The apparent sensing of LPS by LORE seems to be due to contamination of LPS purifications with minute amounts of mc-3-OH-FA. Indeed, purified LORE interacts with mc-3-OH-FA [55].

Thus far, LORE homologs are confined to the Brassicaceae family, hence other undefined receptors are likely to be responsible for sensing LPS, or similar metabolites, in other plant families. In rice (*Oryza sativa*), OsCERK1, the receptor for chitin oligomers and peptidoglycan, also appears to mediate sensing of LPS from several bacterial species, including *P. aeruginosa* [56]. However, whether LPS is the specific ligand of this receptor has not been shown and requires further investigation. Two *A. thaliana* proteins related to LBP, AtLBR-1 and AtLBR-2, were recently discovered and shown to bind LPS. Mutants of *AtLBR-1* and *AtLBR-2* were deficient in some of the typical LPS responses and AtLBR-2 also appears to respond to *P. aeruginosa* LPS by inducing a number of genes related to defence against pathogens [57,58]. Despite these advances in understanding LPS sensing in plants, much work needs to be done to elucidate the species-specific events that lead to induction of plant immune responses.

### 3.2. LPS Stimulates and Inhibits Host-Mediated Bacterial Defences

LPS is an inducer of the complement system, a cascade of proteins that recognizes microbes and induces localized inflammatory responses, phagocytosis, and deposition of the pore-forming membrane attack complex (MAC). The complement cascade can be activated by the classical mannose-binding lectin, as well as alternative pathways [59]. Lipid A, core, and O antigen activate one or more of these pathways but bacteria expressing long O antigen chains are usually more resistant to serum than their O antigen-deficient isogenic mutants [60,61,62,63,64,65,66]. However, specific chain lengths of O antigen have been shown to be important in conferring resistance [67,68,69,70,71,72,73]. Nonetheless, some bacteria are resistant to serum-killing effects in the absence of O antigen, e.g., *Brucella melitensis* [74]. In *P. aeruginosa*, the long but not the very long chains of OSA are necessary for serum resistance, and the total loss of regulation of O antigen chain length in mutant strains defective in the expression of the *wzz* gene results in attenuation in a mouse model of pneumonia [72,73]. Interestingly, a serum-resistant *P. aeruginosa* mutant derived from a serum-sensitive CF isolate displayed an increase in the production of long OSA chains, further supporting the role of chain length in this organism [75]. The exact role of O antigen in conferring serum resistance may vary between organisms, but the activation of MAC (complement proteins C5 to C9) away from the bacterial membrane [76,77,78,79], inefficient convertase formation due to blocking of C3b-factor B binding sites [80], and poor interaction of certain polysaccharides with C3b have all been observed as contributing factors to serum resistance [76,80]. Additionally, the antibody response to bacterial polysaccharides can also be detrimental to the effectiveness of complement-mediated killing. As reported by Wells et al., the serum of some patients with chronic bronchiecstasis were shown to have inhibited killing of *P. aeruginosa* due to increased anti-O antigen IgG_2_ antibody titers. The authors hypothesized that the increase in O antigen-specific IgG_2_ blocked complement deposition or other antibodies from reaching the cell surface [81]. Importantly, this inhibitory effect was correlated with decreased lung function and mirror early studies that found a similar relationship between elevated anti-O antigen IgG_2_ with poor prognosis in CF patients [82,83].

The presence of O antigen also protects bacteria from phagocytosis and has been demonstrated for many organisms [84,85,86,87,88,89,90,91]. Once engulfed, O antigen may facilitate bacterial survival or delay the onset of recognition of immune receptors. This is particularly important for pathogens that replicate intracellularly, such as *Brucella*, which has been shown to delay lysosome fusion with phagosomes and delay apoptosis in an O antigen-dependent manner [92,93]. The opsonization of bacterial surfaces by lectins facilitates microbial killing and clearance by phagocytosis. In the lung, *P. aeruginosa* encounters opsonizing lectins that target LPS, such as surfactant protein A (SP-A) and surfactant protein D (SP-D). *P. aeruginosa* strains that are able to glycosylate pilin with O antigen subunits are more frequently identified in CF isolates compared to those in non-CF isolates [94], and this modification was shown to increase bacterial fitness by providing resistance to opsonization by SP-A and SP-D. When combined with the observations that the lungs of CF patients are typically deficient in SP-A, SP-D, and other LPS-targeting lectins, this points to one possible reason why *P. aeruginosa* is a particularly good pulmonary pathogen [95].

LPS can also stimulate neutrophils to release neutrophil extracellular traps (NETs) that sequester invading pathogens. The current literature suggests that the CF lung is enriched with NETs, and one hypothesis is that this may drive selection for mucoid *P. aeruginosa* (overproduction of the biofilm polysaccharide alginate), a hallmark of chronic infection isolates [96]. Under conditions that mimic those found in the tissues, the release of NETs (so-called “NETosis”) is induced by *P. aeruginosa* LPS, presumably in an O antigen-specific manner [97].

## 4. LPS Influences Bacterial Physiology

### 4.1. OMV Biogenesis and Packaging

Outer membrane vesicles (OMVs) are produced by deliberate blebbing of the Gram-negative OM and are enriched with various biomolecules. OMVs have been reported to play important roles in cell–cell communication, antibiotic resistance, biofilm structure, and long-range delivery of public goods, toxins, and virulence factors [98]. Not surprisingly, since these vesicles are derived from LPS-rich membranes, OMV production is intricately linked to LPS structure. A major contributor to OMV biogenesis in *P. aeruginosa* is the production of the *Pseudomonas* Quinolone Signal (PQS). PQS is one of the molecules of the complex *Pseudomonas* quorum sensing circuit, which regulates *P. aeruginosa* group behaviours, virulence factor production, and biofilm formation. The highly hydrophobic PQS is exported to the OM, promoting its own excretion in OMVs by interacting with Lipid A acyl chains and phosphates, which induces membrane curvature [99,100,101,102]. Remodeling of lipid A in response to environmental cues also influences OMV biogenesis. Recent experiments in *S.* Typhimurium revealed that lipid A species modified with l-4-aminoarabinose and phosphoethanolamine were less likely to be incorporated into OMVs, whereas lipid A that was hepta- or penta-acylated were enriched in OMVs. This differential incorporation correlates with the geometry of the lipid species (more cylindrical versus conical, respectively) and consequently their propensity to induce membrane curvature and vesiculation [103,104].

O antigen also plays a role in OMV biogenesis but is poorly understood. First, it was noticed by Kadurugamuwa and Beveridge [105] that naturally occurring *P. aeruginosa* OMVs contained the anionic OSA, but not the neutral CPA, leading them to propose that charge repulsion of the O antigen chains induces membrane curvature and membrane budding. A similar observation was made in *Porphyromonas gingivalis*, wherein the anionic A-LPS was enriched in OMVs compared to the neutral O-LPS. The proteins carried by OMVs of *P. aeruginosa* and *P. gingivalis* are altered in the absence of the anionic O antigens, suggesting they are involved in the selective protein sorting process [106,107]. *P. gingivalis* proteins are linked to A-LPS through the type IX secretion system and may be a means of directing this sorting [108], but would be by other means in *P. aeruginosa*, which lacks this system. The presence or absence of O antigen also has implications for the kinetics of entry into host cells. OMVs derived from O antigen^+^ *E*. *coli* enter the cells faster and through lipid raft endocytosis whereas those from O antigen^−^ *E. coli* are endocytosed slower through clathrin-coated pits [109].

### 4.2. The Role of LPS in Planktonic and Biofilm Modes of Growth

O antigen is necessary for effective swimming and swarming motility in many bacteria [110,111,112,113,114,115,116,117,118,119,120], which has been demonstrated in genetic studies that investigated the effect on motility when genes involved in various steps of the O antigen synthesis and assembly pathway are deleted. Our group reported the phenotypes of several such mutants in *P. aeruginosa*. Firstly, deletion of the protein responsible for attaching O antigen to the core only expresses lipid A-core on the surface and results in the loss of swimming and swarming motility due to a substantial decrease in flagella assembly [121]. Secondly, deletions in *P. aeruginosa* genes that result in a truncated core region (and the blocking of attachment of O antigen) are defective in swarming and swimming on semi-solid media, but this is not due to defects in flagella synthesis or function [122]. The defect is apparently due to increased cell hydrophobicity, leading to stronger cell–cell association, which was supported by further studies of cell physical properties using atomic force microscopy [122,123,124]. Similarly, O antigen may mediate surface translocation by acting as a surfactant or by increasing the “wettability” of the cell surface [118].

Bacteria often grow as biofilms, which are complex communities encased in an intricate polymeric structure composed of polysaccharides, DNA, proteins, and lipids that protect the cells from external stress. In contrast to the motile planktonic mode of growth, biofilms are usually attached to biotic or abiotic surfaces and are a major cause of persistent infection. *P. aeruginosa* is a model organism for studying biofilms. In fact, it was the first organism implicated in a medically associated biofilm when cell aggregates were observed in the sputum of CF patients [125]. The O antigen is linked to biofilm formation, although, whether biofilm production is positively or negatively affected by the presence of O antigen varies between bacterial species and may be influenced by the surface tested [126].

The *P. aeruginosa* LPS structure is highly dynamic during biofilm growth, and the production of different chemotypes may be beneficial for certain stages of biofilm development. Overall, the literature suggests that CPA is more important than OSA in establishing robust biofilms. In vitro experiments showed that as *P. aeruginosa* adapts to the biofilm mode of growth, the production of OSA but not CPA is decreased [127]. However, strong selective pressure for an OSA-deficient phenotype will eventually lead to mutations in OSA biosynthesis [128]. In the CF lung, OSA expression is usually lost by acquiring mutations in the biosynthetic gene clusters, while CPA expression is more stable [129]. One study by our group showed that mutations that result in the loss of O antigen, but leave an intact core, produce biofilms with a similar structure and biomass. However, differences were noted in a mutant strain (Δ*rmd*) where only the synthesis of CPA, but not OSA, was disrupted. Between 16 h and 48 h of biofilm growth, the density of cells and exopolysaccharides was gradually reduced, suggesting a defect in biofilm maturation [107]. These results were substantiated in another study that observed the restoration of biofilm maturation when CPA synthesis was rescued in a CPA-deficient isolate, PA14 [130]. The importance of CPA in biofilms is also demonstrated by its link to the secondary messenger cyclic-di-GMP, the so-called “master regulator”, that induces the physiological changes necessary to switch from motile planktonic growth to sessile biofilm growth. The CPA O antigen chain length was decreased by a cyclic-di-GMP-responsive methyltransferase, WarA, suggesting that CPA modification may be involved in the switch to a biofilm growth [131]. Interestingly the rhamnose-rich CPA is similar to the O antigens of many phytopathogens [132,133,134], raising the possibility that CPA evolved to allow *P. aeruginosa* to develop biofilms on plants and soil. The role of OSA in biofilm biogenesis is less clear, but one proteomics study presented intriguing data to indicate that the proteins that regulate OSA length are overproduced upon attachment to a glass wool surface, suggesting a role that OSA plays in the early stages of biofilm development [135]. Once biofilms are established, the production of long OSA chains may no longer be necessary. In line with this, very long OSA chain lengths are downregulated in mucoid *P. aeruginosa* (a hallmark of chronic infection) [136]. It is noteworthy that the link between OSA and biofilm development has mostly been studied in PAO1 (serotype O5). Since each serotype has a unique polysaccharide structure that will have different physiochemical properties, how OSA influences biofilm development in other serotypes should be explored.

## 5. Antimicrobials Target LPS

### 5.1. Phages and Pyocins

The extension of LPS into the extracellular milieu makes it a prime receptor for many bacteriophages. LPS is therefore integrally linked to the phage life cycle and bacteria experience strong selective pressure by phage predation to remodel their LPS. Some phages may recognize the O antigen or the core OS, either exclusively or in addition to outer membrane proteins. Accordingly, phages can be highly specific for a given O antigen serotype, or can have a broader host range if they recognize more conserved constituents of LPS [137]. A number of LPS-specific phages that target *P. aeruginosa* have been described in the literature as well as phage-resistant mutants arising from mutations in LPS biosynthesis genes [138,139,140,141,142,143,144,145,146,147]. In other cases, phage resistance may arise when temperate bacteriophages encode proteins that modify the O antigen, conferring resistance to superinfection [148]. The *P. aeruginosa* temperate bacteriophage D3 encodes a peptide that inhibits the host O antigen polymerase, allowing a separate phage polymerase to dominate and produce O antigen with a β linkage instead of an α linkage between O units, resulting in seroconversion [141,149,150]. Clearly, phage predation can influence the LPS phenotype of bacteria and drive O antigen diversity. Understanding phage–LPS interactions is important because LPS-specific phages often encode enzymes that degrade or modify the O antigen in order to gain access to the cell membrane. These enzymes may be useful in developing novel narrow-spectrum antimicrobial therapies [151]. For instance, Olszak et al. showed that a polysaccharide lyase from phage LKA1 degrades *P. aeruginosa* serotype O5 OSA, and this sensitized bacteria to serum complement, reduced virulence in a wax moth larvae infection model, and disrupted biofilms [147].

Bacteriocins are proteinaceous antibiotics produced by bacteria for intra- or inter-species killing. *P. aeruginosa* produces a number of bacteriocins, termed S- R-, and F-pyocins. The S-pyocins are analogous to the colicins produced by *E*. *coli*: They are proteins that hijack outer membrane proteins to gain access to the cell and exert their killing effects via a toxin domain. Producers of S-pyocins are protected from self-killing by immunity proteins that block the cytotoxic activity. R- and F-pyocins (also called tailocins) evolved from contractile and flexible phage tails, respectively, and kill by puncturing the bacterial membrane and inducing depolarization [152]. The lectin-like bacteriocins (L-pyocins; Llb) are comprised of one or two monocot mannose-binding lectin domains (MMBL) and may kill at the OM surface by blocking the function of BamA, a protein of the β-barrel assembly machinery [153,154]. All three classes of pyocins have been shown to interact with different LPS constituents. Some S- and L-pyocins bind CPA to target their other membrane receptors, and the loss of CPA decreases killing efficiency by these bacteriocins [155,156]. In contrast, the R-pyocins exclusively recognize LPS and do not have secondary OMP receptors. Using defined LPS mutants, different subtypes of R-pyocins were determined to recognize different core constituents [157,158]. For instance, the terminal GlcIV of the uncapped glycoform is part of the receptor for the R3-pyocin since strains lacking this residue are resistant to R3-mediated killing [158]. The recent structure of N-terminally truncated R2 pyocin suggests that a “foot domain” binds the core and that patches of mutations within specific loops of this domain drive specificity. Additionally, a distal “head domain” may bind O antigen [159]. Characterization of these LPS recognition domains may allow researchers to alter these killing particles to target a strain of choice and develop new antimicrobials. Importantly, the presence of LPS capped with OSA can protect bacteria from R pyocins, presumably by blocking access to the core [157]. In fact, the loss of O antigen due to mutations acquired during biofilm growth can result in sensitivity to self-produced R pyocins and a so-called “culture-impaired” phenotype, i.e., a drastically reduced ability to grow in liquid media [128]. O antigen likely also protects *P. aeruginosa* from the killing effects of S pyocins since studies in enterics have shown that the O antigen chain length and density are important factors in protecting them against colicins [160,161,162]. Interestingly, since a high proportion of CF isolates are susceptible to at least one subtype of R pyocin, the use of pyocin cocktails could potentially be viewed as highly targeted therapeutics to treat chronic infections in CF patients [163]. Indeed, the efficacy of a number of pyocins has been demonstrated in in vivo animal models [164,165,166,167].

Pyocins apparently play complex roles in establishing *P. aeruginosa* communities. The presence of R pyocins was shown to result in increased attachment and biofilm formation in susceptible strains at certain concentrations, but the mechanisms have yet to be determined [168]. Could pyocin-mediated killing drive changes to LPS that affect biofilm development (see above), or is this the result of a general stress response? In a separate study, R pyocins produced by one CF strain were necessary to outcompete another in a biofilm competition assay. When both strains lacked the ability to produce R pyocins, or when isolates producing the same R-pyocin subtype were grown together, the bacteria coexisted as a patchwork (adjacent communities) of individual strains [169]. Hence, these studies show that LPS-mediated pyocin susceptibility drives changes in the biofilm architecture and community, which may have downstream effects on disease outcomes.

### 5.2. LPS-Mediated Antibiotic Resistance

A myriad of oral, intravenous, and inhaled antibiotics are used to treat CF patients infected with *P. aeruginosa* and these include both monotherapies and combined antibiotic treatments [170]. Among these antibiotics are colistin (polymyxin E) and tobramycin (an aminoglycoside), whose efficacy is directly related to the LPS structure. In light of the increasing number of infections caused by multi-drug resistant Gram-negative bacteria, polymyxins have re-emerged in the clinic as last resort antibiotics. Polymyxins are cationic antimicrobial peptides (CAMPs) that target GNB through electrostatic interactions with lipid A and core phosphates, which is necessary for the self-mediated uptake of these antibiotics through the OM [171]. Accordingly, GNB defend against polymyxins by modifying the charge of their LPS through addition of positively charged moieties to lipid A phosphates. The best-described modifications in the literature are addition of l-4-aminoarabinose (l-Ara4N) and phosphoethanolamine (PEtN). These modification systems are controlled by complex networks of two-component regulators that sense magnesium, iron, zinc, cationic antimicrobial peptides, and pH [172]. The proteins for the synthesis and transfer of l-Ara4N are encoded chromosomally by the *arn* operon while PEtN addition is encoded chromosomally by *eptA* or the plasmid-borne *mcr*, which has garnered global concern due to its ability to mobilize colistin resistance [173]. *P. aeruginosa* can modify lipid A with both l-Ara4N and PEtN, but only l-Ara4N confers polymyxin resistance [174,175]. Although other mutations may play a role in polymyxin resistance, in vitro evolution studies point to the primary role of aminoarabinsoylation in establishing a trajectory towards high-level resistance. Firstly, high-level colistin resistance does not evolve in the absence of a functional *arn* operon [176]. Secondly, mutations in the two component systems controlling expression of the *arn* operon typically evolve first, and are necessary for synergistic interactions with mutations in other LPS biosynthesis genes, namely those involved in lipid A and core biosynthesis [177,178]. The acylation pattern of lipid A can also confer polymyxin resistance. PagL expression, which removes the 3-*O*-linked acyl chains from lipid A in the OM, is induced by polymyxin B and increases resistance to this CAMP only in an already resistant strain that constitutively aminoarabinosylates its lipid A. PagL-mediated resistance is due to decreased penetration of polymyxin B penetration through the OM, owing to the fewer available hydrophobic interactions with an underacylated lipid A [179]. Colistin insensitivity may also arise without modification of LPS. Yokota et al. showed that an inoculation effect can increase the MIC of colistin, which was attributed to the release of LPS either from dead cells or from OMVs [180]. These results agree with those of Manning and Kuehn, who showed that OMVs could protect bacteria by sequestering AMPs [181].

Since aminoglycosides also interact with the OM and enter the bacterial cell through self-promoted uptake, aminoarabinosylation of lipid A similarly confers resistance to these antibiotics. Importantly, the chelation of divalent cations and acidification of biofilms by extracellular DNA induces the *arn* operon and increases the aminoglycoside resistance of *P. aeruginosa* [182,183]. Additionally, loss of O antigen side chains is correlated with resistance to aminoglycosides, possibly by reducing binding to the cell surface [184] and membrane permeabilization [185].

### 5.3. New Classes of Antibiotics Target the LPS Biosynthesis Machinery of P. aeruginosa

New strategies are desperately needed to treat *P. aeruginosa* and other Gram-negative pathogens. Since LPS is essential to almost all GNB, the biosynthesis pathways are attractive targets for antimicrobial development, especially since these pathways often use substrates not found in humans. Inhibitors of the first committed step of lipid A biosynthesis, LpxC, have been designed with both broad and narrow spectrum antimicrobial activity (reviewed in [186]). Among these antimicrobials, a *Pseudomonas*-specific inhibitor developed by Achaogen was the only one to advance to Phase 1 clinical trials [187]. However, this compound was abandoned due to dose-limiting cardiovascular toxicity. Further development of this compound yielded several new leads, but the cardiovascular toxicity and narrow therapeutic window re-emerged in pre-clinical animal models [188]. Additionally, the potential for gaining resistance to these compounds was deemed too great to proceed [187]. Unfortunately, Achaogen filed for bankruptcy in April 2019, so the future development of these compounds remains uncertain [189].

A macrocyclic peptidomimetic antibiotic developed by Polyphor, termed Murepavadin (POL7080), has specific activity against *Pseudomonas* spp. This antibiotic targets LptD, an OMP of the LPS transport machine that, along with LptE, transports LPS from the periplasmic side of the OM to the outer leaflet [190]. The specificity of the antibiotic is due to targeting of a region of the periplasmic domain of LptD that is unique to pseudomonads [191]. Intravenous murepavadin has undergone Phase II clinical studies in patients with ventilator-associated bacterial pneumonia and non-cystic fibrosis associated bronchiecstasis but Phase III trials were halted after higher than expected renal toxicity was observed. In a 4 September 2019 news release, Polyphor has indicated that an inhalable murepavadin for treatment of *P. aeruginosa* infection in cystic fibrosis patients is expected to begin clinical trials in 2020 [192]. Concerningly, resistance to this compound may already exist and/or drive resistance to other antimicrobials. Romano et al. reported that resistance (4–32-fold change in MIC) to POL7080 can develop through *pmrB* mutations that upregulate the *arn* operon, resulting in decreased binding to the cell surface and the development of cross-resistance to colistin [193].

## 6. Concluding Remarks

**Figure 3:**
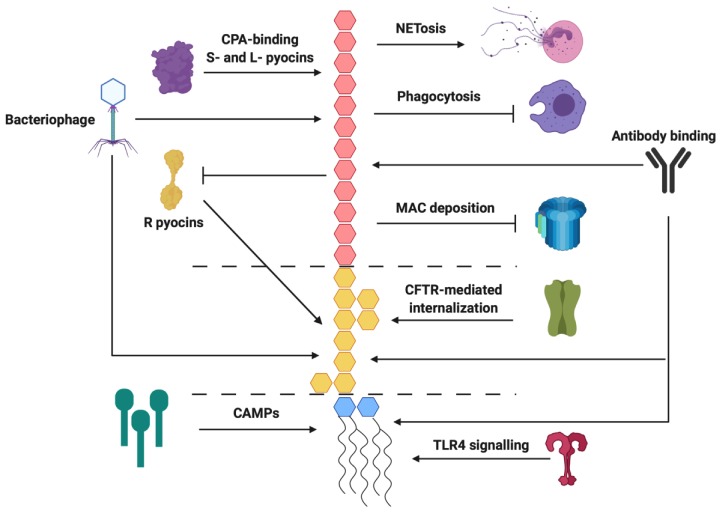
Summary of relevant *P. aeruginosa* O antigen, core, and lipid A interactions with antimicrobials and host defences. Arrows indicate binding of, or activation by, a specific LPS region while the flat-headed arrows indicate inhibition. The O antigen is coloured red, the core is coloured yellow, and lipid A is coloured blue.

In this review, we have described how LPS contributes to the pathogenesis of *P. aeruginosa* by (i) interacting with host receptors, (ii) inhibiting host defence systems, (iii) influencing the biogenesis of biofilms and OMVs, and (iv) mediating resistance to antimicrobials (Figure 3). Although the role of the lipid A, core, and O antigen moieties in these processes have been extensively studied, a comprehensive understanding of the interplay between LPS and pathogenesis will require further research. For instance, interactions of LPS with host proteins has been the focus of many studies, but recent research suggests that glycan–glycan interactions between bacteria and their hosts may be more relevant than previously realized [194,195,196]. The advent of new glycan arrays could facilitate the screening and discovery of novel LPS interactions and yield new insights into pathogenic processes [194,197,198].

The LPS glycoforms expressed on the cell surface can influence the biofilm mode of growth, yet the underlying mechanisms are poorly understood. Although differences in cell adhesion/cohesion may explain the propensity of a strain to form biofilms [122,123,124], the possibility that LPS may interact with matrix material also needs to be investigated. For instance, could CPA promote biofilm maturation by coordinating protein or polysaccharide components within the matrix? The role of LPS in biofilm development has mostly been studied on short time scales, under laboratory conditions. Since changes to LPS occur over the course of a chronic infection, the consequences of these changes on biofilm physiology should also be studied and with more clinically relevant systems. Additionally, other OSA polysaccharides may have more pronounced roles in biofilm development and should be investigated. O antigen also influences the selective packaging of cargo into OMVs, which are another component of biofilms. How O antigen is involved in this process is not understood and represents a significant gap in our knowledge. Unraveling this mechanism and characterizing the proteins that are deliberately packaged into OMVs may give clues to the function of OMVs within planktonic and biofilm communities.

A comprehensive understanding of LPS biosynthetic enzymes, gene regulatory pathways, and structure will be essential to developing new antimicrobials against *P. aeruginosa* and other Gram-negative pathogens. Although some promising compounds targeting LPS biosynthesis and transport have been developed, overcoming the development of resistance is a major hurdle that must be considered. The generation of therapeutics that inhibit the Lipid A modification pathways will be indispensable to mitigating CAMP resistance, while the continued characterization of “natural” antimicrobials that use LPS as a receptor, such as phages and bacteriocins, may be an effective way to develop targeted therapeutics. Finally, although LPS-based *P. aeruginosa* vaccines had only limited success in the past [199], this may still prove to be a promising prophylactic treatment since the use of OMVs and recombinant bacterial glycosylation pathways to produce glycoconjugates has made these vaccines safer, more effective, and cheaper to produce [200].
